# Supplementary material for: Inducing mitophagy in diabetic platelets protects against severe oxidative stress
Source: EMBO Mol Med. 2016 May 24;8(7):779–95. doi: 10.15252/emmm.201506046 (PMC4931291; doi:10.15252/emmm.201506046)

Appendix Table of Contents

1. Appendix Figure legends S1-S13 and Appendix Table S1 and S2

2. Appendix Figures S1-S13

1. Appendix Figure legends S1-S13 and Appendix Table S1 and S2

Appendix Figure S1. **Western blot analysis of oxidative adducts**

Oxidative adducts (3-nitrotyrosine (3-NT), aldehyde adducts (4-HNE), carbonyl derivatives (dinitrophenyl (DNP)-derivatized carbonyl)) and polyubiquitination in pooled HC (n=4) and pooled DM (n=8) platelet protein lysates. GAPDH was used as the loading control. Quantification analysis of each oxidative adducts were performed using the Image J program.

Appendix Figure S2. **High magnification EM of platelet from HC and DM samples.**

EM of platelets from HC and DM patients demonstrating increased vacuolation in DM platelets.

Appendix Figure S3. Mitophagy is induced in DM platelets.

A. Triple staining for CoxIV, LC3, and LAMP1 (lysosome) in HC and DM platelets. A representative enlarged single platelet is shown. Arrow indicates positive signal for LC3 and LAMP1. Graph indicates colocalization between LC3, LAMP1 and CoxIV signal. The y-axis indicates fold change of colocalization between LC3 and LAMP1 in the mitochondria signaling area (*$p<0.05$ vs. HC). Signal intensity of each group was converted to fold compare with HC values**.**

B. Representative Western blot analysis showing increased Parkin and LC3 protein expression in DM platelet mitochondria fraction.

C. Immuno-EM of DM samples (DM) demonstrating multiple clusters and colocalization between LC3 and Parkin. Red arrow indicates immunogold-labeled LC3 antibody (10 nm) and black arrow indicates immunogold-labeled Parkin antibody (5nm). Black bars in the inset represent 200 nm.

D. Triple staining for CoxIV, LC3, and Parkin (mitophagy) in HC and DM platelets. A representative enlarged single platelet is shown. The arrows indicate positive signal for LC3 and

Parkin. Graph indicates colocalization between LC3, Parkin, and CoxIV signal. The y-axis indicates fold change of colocalization between LC3 and Parkin in mitochondria (CoxIV) (**$p<0.01$ vs. HC). Signal intensity of each group was converted to fold and compared with HC values.

Appendix Figure S4. Western blot analysis of Bnip3/Nix in HC and DM platelets.

A. Representative Western blot analysis of Bnip3/Nix, Parkin and LC3II in HC (#7-9) and DM (#19-26) platelets.

B. Graph indicate quantification Bnip3/Nix, Parkin and LC3II in HC (#7-9) and DM (#19-30) platelets. (**$p<0.01$, *$p<0.05$ vs. HC). GAPDH served as a loading control.

Appendix Figure S5. qPCR analysis of mitochondrial content in human and mice DM platelets.

A. Representative qPCR analysis of NADH dehydrogenase subunit 1 (ND1) and 16sRNA in human DM platelets (HC=3 and DM=3). 18sRNA was used for normalization. (**$p<0.01$, *$p<0.05$ vs. HC). qPCR primers used for human : ND1 F: 5′-ACG CCA TAAAAC TCT TCA CCA AAG-3′ ,ND1 R: 5′-TAGTAG AAG AGC GAT GGT GAG AGC TA-3′ ,16s RNA F: 5’-GAACCAGACGAGCTACCTAAG-3’ and 16s RNA R: 5’-GGTTTGTCGCCTCTACCTATAAA-3’

B. Representative qPCR analysis of NADH dehydrogenase subunit 1 (ND1) in mouse DM platelets (HC=3 and DM=3). 18sRNA was used for normalization. (**$p<0.01$, *$p<0.05$ vs. HC).

qPCR primers used for mouse platelets: ND1 F: 5′- TGCACCTACCCTATCACTCA-3′ and ND1 R: 5′-GGCTCATCCTGATCATAGAATGG -3′

Appendix Figure S6. Western blot analysis of Ulk activation and mTOR downstream targets in HC and DM platelets.

- **A.** Representative Western blot analysis of phosphorylation of Ulk (S757) and total Ulk in HC (#4-6) and DM (#9-18) platelets. Graph indicates quantification of phosphorylation of Ulk (S757) and total Ulk (**$p<0.01$, *$p<0.05$ vs. HC). GAPDH served as a loading control.
- **B.** Representative Western blot analysis of phosphorylation of p70S6K and S6 in HC (#7-9) and DM (#19-26) platelets. Graph indicates quantification of phosphorylation of p70S6K and S6 in HC (#7-9) and DM (#19-30) platelets. (**$p<0.01$, *$p<0.05$ vs. HC). GAPDH served as a loading control.

Appendix Figure S7. p53 activity does not affect ROS induced autophagy.

- **A.** HC platelets were treated with H_2O_2 to induce autophagy and treated with or without PFT-α (p53 inhibitor, 10 and 20μM) to assess for inhibition of autophagy. This Western blot analysis is representative of three independent experiments.
- **B.** Quantification of the three independent experiments is provided at right. (**$p<0.01$, *$p<0.05$ vs. HC or H_2O_2 group, n=3 for each group.
- **C.** Triple staining with Mitotracker, CoxIV, and LC3 in HC and H_2O_2 with or without 10μM PFT-α treated platelets using confocal microscopy.

Appendix Figure S8. Mitophagy induced in human platelet and maybe protective

A. Triple staining for CoxIV, LC3, and Parkin in HC platelets. Treatments were performed with high glucose (HG) and/or CCCP. Enlarged areas are provided showing areas of colocalization (CCCP treatment, three arrows in proximity). All images are representative of 5 images taken randomly and repeated three times in independent experiments.

B. Graph indicates colocalization between LC3, Parkin and CoxIV signal. The y-axis indicates fold change of colocalization between LC3 and Parkin in the mitochondria (CoxIV). Signal intensity of each group was converted to fold and compared with HC values (***$p<0.01$* vs. HC or HG group).

C. $\Delta\Psi m$ and platelet apoptosis were measured by flow cytometry analysis in HC, HG, HG with H_2O_2, or HG with H_2O_2 and 3MA using human platelets. $\Delta\Psi m$ was detected using TMRM and apoptosis level was assessed with Annexin-V (PS externalization). Graph indicates the percentage of TMRM negative cell in the total cell population (***$p<0.01$*vs. HG or HG with H_2O_2, NS means no significance).

D. Graph indicates the percentage of Annexin V positive cell in total cell population (***$p<0.01$* vs. HG with H_2O_2, NS means no significance).

Appendix Figure S9. Human platelet mitophagy protects in DM.

The recognized autophagy inhibitor (20μM Spautin-1 for 2h) was used to treat DM platelets and compared to HC assessing for phosphorylated p53, p53, and LC3I/II. Graph indicate quantification of phosphorylation of p53 (S15) and LC3II (**$p<0.05$* vs. HC). GAPDH served as the loading control.

Appendix Figure S10. Mitophagy reduces platelet aggregation.

Platelet suspensions were incubated with CCCP for 1hr. Platelet aggregation was monitored at 37°C with constant stirring (1200 rpm) in a dual-channel lumi-aggregometer (model 700; Chrono-Log). Platelet aggregation was measured as the increase in light transmission for 10 minutes, starting with the addition of 2 μl of 1 mg/ml collagen (Chrono-Log) into 500 μl as a proaggregatory stimulus; the final concentration was 4 μg/ml. The maximum aggregation was expressed as a percentage of maximum light transmission, with nonstimulated PRP being 0% and PPP 100%.

A. HC platelet suspensions were incubated with or without CCCP for 1hr. Percentage of light transmission was measured in platelet suspensions under DMSO (black line) and CCCP (Blue line) in response to 4 μg/ml collagen for 10 minutes (*n* = 4 in each group).

B. Western blot analysis and quantitation analysis using 4 μg/ml collagen treatment samples with or without CCCP.

C. Graph indicates quantification of LC3II. (**p<0.05*). GAPDH served as a loading control.

Appendix Figure S11. Platelet apoptosis in PINK1 knockout mouse platelets.

A. Western analysis in WT and PINK -/- demonstrating the inability to induce LC3 above basal levels despite the addition of H_2O_2. Shown graphically are the LC3II expression level (**p<0.05*, n=3)

B. ΔΨm and platelet apoptosis were measured by flow cytometry analysis in WT, PINK1-/-, WT treated with H_2O_2 or PINK1-/- treated with H_2O_2. ΔΨm was detected using TMRM (***p<0.01,* n=3 for each group)

C. Apoptosis level was assessed with Annexin-V (PS externalization). Graph indicates the percentage of Annexin V positive cell in the total cell population (***p<0.01*, **p<0.05*, NS no significance, n=3 for each group)

D. ΔΨm and platelet apoptosis were measured by flow cytometry analysis in WT, Parkin -/- , PINK1-/-, WT treated with H_2O_2 or Parkin-/- treated with H_2O_2 or PINK1-/- treated with H_2O_2 (0.5 or 1mM). ΔΨm was detected using TMRM (***p<0.01,* n=3 for each group)

E. Apoptosis level was assessed with Annexin-V (PS externalization) by flow cytometry analysis in WT, Parkin -/- , PINK1-/-, WT treated with H_2O_2 or Parkin-/- treated with H_2O_2 or PINK1-/- treated with H_2O_2 (0.5 or 1mM). Graph indicates the percentage of Annexin V positive cell in the total cell population (***p<0.01*, **p<0.05*, NS no significance, n=3 for each group)

Appendix Figure S12. Western blot analysis of p53, JNK, LC3I/II in NAC treatment.

A. In vivo treatment of WT vs DM mice with NAC (15mg/kg I.P. for 1 hour) followed by CellRox detection of ROS and flow cytometry. (**$p<0.01$)

B. $\Delta\Psi m$ (TMRM) and platelet apoptosis (Annexin-V) were measured by flow cytometry analysis in WT, DM and DM with NAC as described in Panel A. (*$p<0.05$)

C. Western blot analysis of JNK, LC3I/II in HC, DM and DM platelets treated with NAC. GAPDH was used as the loading control. Graph indicate quantification of phosphorylation of p53 (S15), phosphorylation of JNK and LC3II (**$p<0.01$, *$p<0.05$ vs. HC). GAPDH served as a loading control.

D. In vivo treatment of WT vs PINK-/- DM mice with NAC (15mg/kg I.P. for 1 hour) followed by CellRox detection of ROS and flow cytometry. (**$p<0.01$)

E. WT vs PINK-/- DM mice platelet apoptosis (Annexin-V) were measured by flow cytometry analysis in WT, DM and DM with NAC as described in Panel A. (*$p<0.05$)

Appendix Figure S13. **Assessment of purity of prepared platelet preparation**

To assess for washed platelet contamination by different blood cells, we used the monocyte/macrophagy marker CD14 and the erythrocyte marker CD235a for Western blotting. Shown is a representative Western analysis.

Appendix Table S1: Clinical characteristics of healthy control and patients with type 2 DM

	Healthy control	T2DM
	n=8	n=66
Age (years ± SD)	41.5±6.3	57.5±12.6
Gender (Males/Females)	8/0	40/26
BMI (kg/m^2 ± SD)	21.8±2.0	34.1±8.6
Blood glucose (mg/dL ± SD)	103.8±4.7	170.7±66.8
HbA1c (% ± SD)	5.2±0.3	7.2±1.8
Systolic blood pressure (mmHg ± SD)	114.1±5.8	134.6±14.7
Diastolic blood pressure (mmHg ± SD)	77.4±3.5	74.7±12.7
Hypertension (%)	0/8 (0%)	47/66 (71.2%)
CAD (%)	0/8 (0%)	17/66 (25.8%)
Medications (%)		
Aspirin	0/8 (0%)	32/66 (48.5%)
Clopidogrel	0/8 (0%)	3/66 (4.5%)
Warfarin	0/8 (0%)	4/66 (6.1%)
Statin	0/8 (0%)	41/66 (62.1%)
Beta-blocker	0/8 (0%)	30/66 (45.5%)
Angiotensin converting enzyme inhibitor	0/8 (0%)	26/66 (39.4%)
Angiotensin receptor blocker	0/8 (0%)	14/66 (21.2%)
Insulin	0/8 (0%)	30/66 (45.5%)
Metformin	0/8 (0%)	21/66 (31.8%)
Other anti-diabetic drugs	0/8 (0%)	8/66 (12.1%)
Diuretic	0/8 (0%)	21/66 (31.8%)

Appendix Table S2: Antibody information

Company	Target	Cat#	Lot#	Clone #	Species	Titer
Cell Signaling	pATK	4060S	16		Rabbit	1:1000
	AKT	2967	11		Rabbit	1:1000
	ATG3	3151P	2		Rabbit	1:1000
	ATG7	8558		D12B11	Rabbit	1:1000
	ATG12	4180		D88H11	Rabbit	1:1000
	Beclin1	5495s	2	D40C5	Rabbit	1:1000
	BNIP3L/NIX	12396S	1		Rabbit	1:1000
	Cleaved Caspase3	9661s	43	D175	Rabbit	1:1000
	Cytochrome C	4272S	6		Rabbit	1:1000
	GAPDH	2118L	8		Rabbit	1:1000
	pJNK	4671S	7		Rabbit	1:1000
	JNK	9258S	9		Rabbit	1:1000
	LAMP1	9091S	2		Rabbit	1:1000 (WB)<br>1:300 (IF)
	PINK	6946P		D8G3	Rabbit	1:1000
	Parkin	4211S	4		Mouse	1:1000
	pp53(S15)	9284			Rabbit	1:1000 (WB)<br>1:500 (IF)
	P53	2524			Mouse	1:1000
	pmTOR(S2448)	2971S	20		Rabbit	1:1000
	mTOR	2972S	6		Rabbit	1:1000
	pULK(S317)	12753S	1		Rabbit	1:500
	ULK	4773S	2		Rabbit	1:500
	Ubiqitin	3936S	9		Mouse	1:1000
	pS6	5364S	6		Rabbit	1:1000
	S6	2217			Rabbit	1:1000
	pp70S6K1	9205L	12		Rabbit	1:1000

	p70S6K1	9202L	14		Rabbit	1:1000
Abcam	LC3	Ab48394			Rabbit	1:500
	Parkin	Ab15954			Rabbit	1:500
	CD14	Ab133335			Rabbit	1:1000
	Glycophorin	Ab129024			Rabbit	1:1000
	Malondialdehyde	Ab27647			Rabbit	1:1000
	4 Hydroxynonenal	Ab46545			Rabbit	1:1000
Cosmo	LC3			LC3 1703	Mouse	1:100 (IF)
Santa cruze	Cox4	SC-69360		G-20	goat	1:100 (IF)
	Tubulin					
Sigma	B-actin	A5316		AC-74	mouse	1:5000

Appendix Figure S1

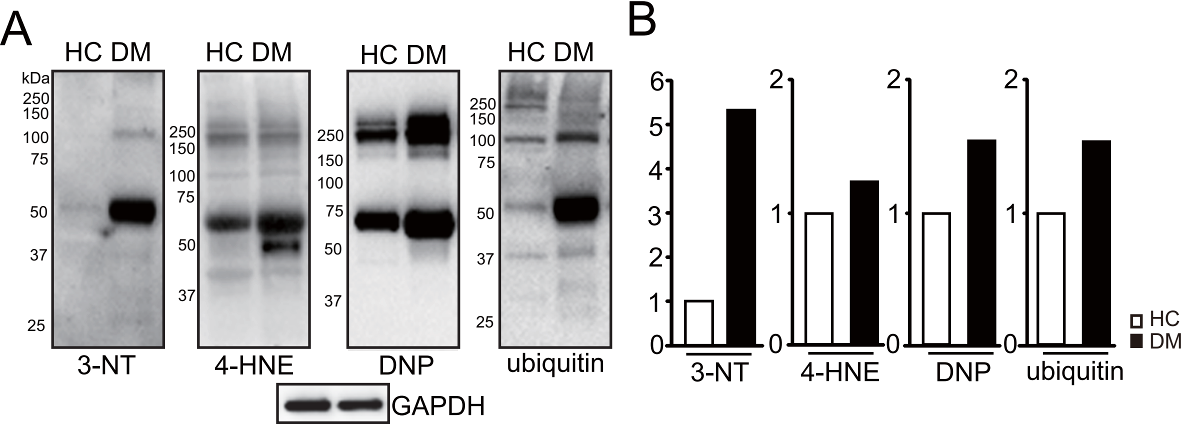

Appendix Figure S2

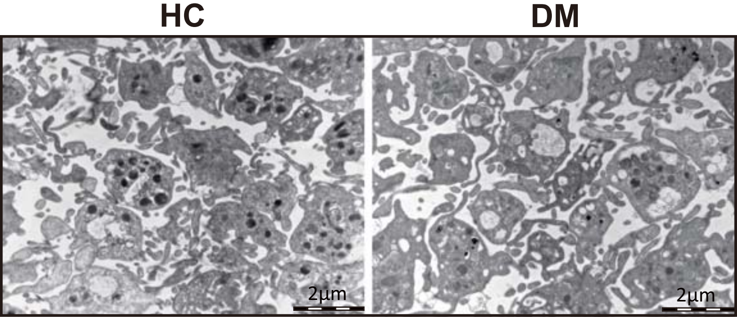

Appendix Figure S3

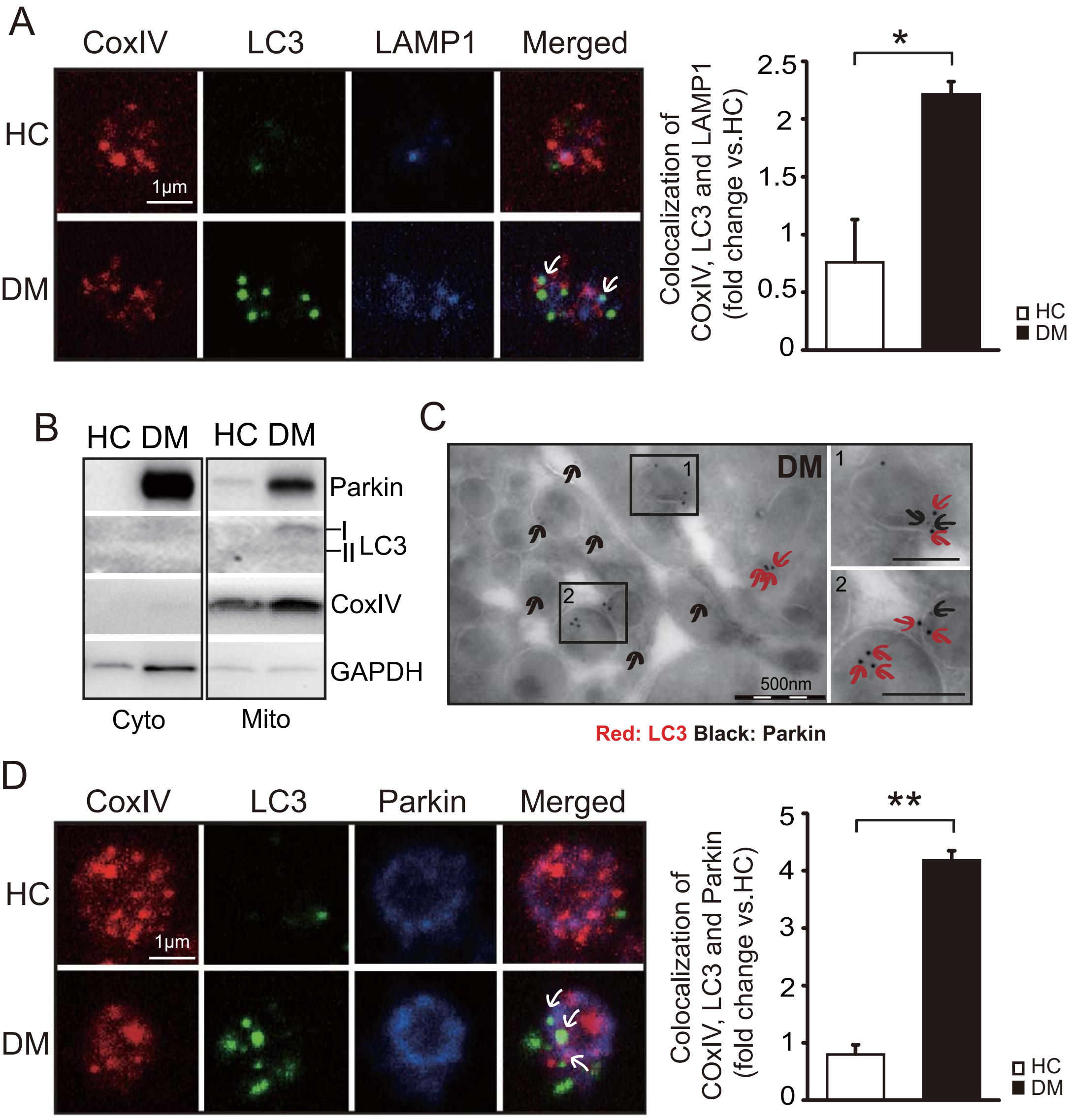

Appendix Figure S4

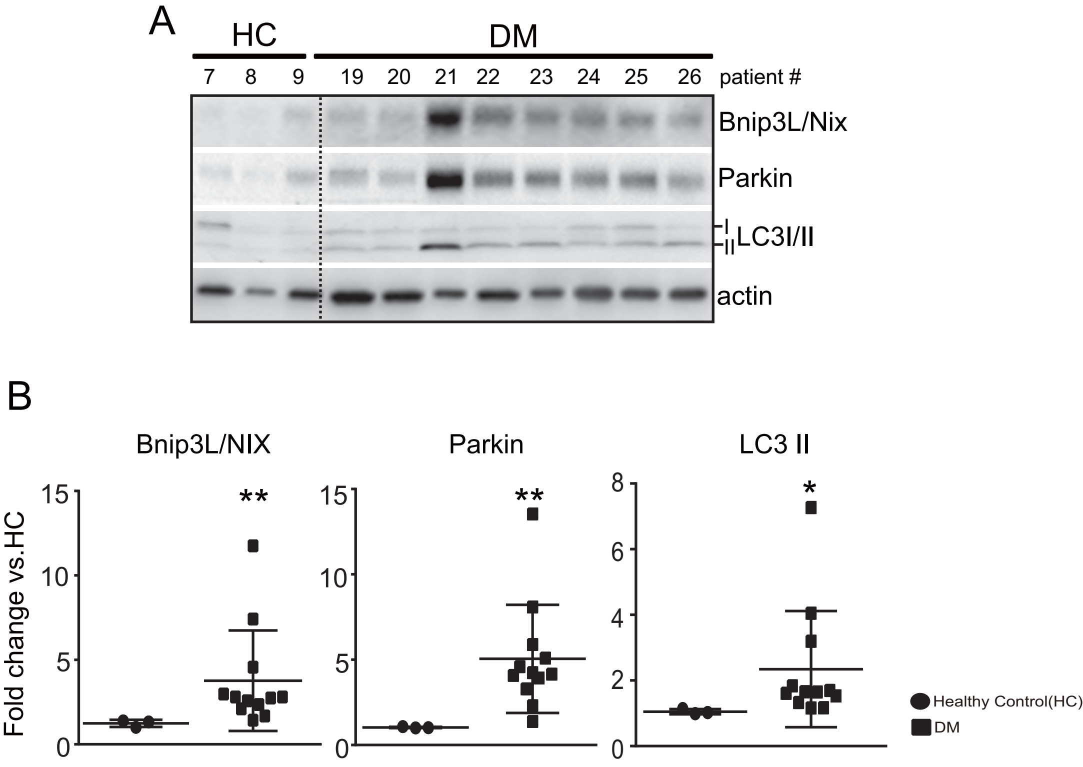

Appendix Figure S5

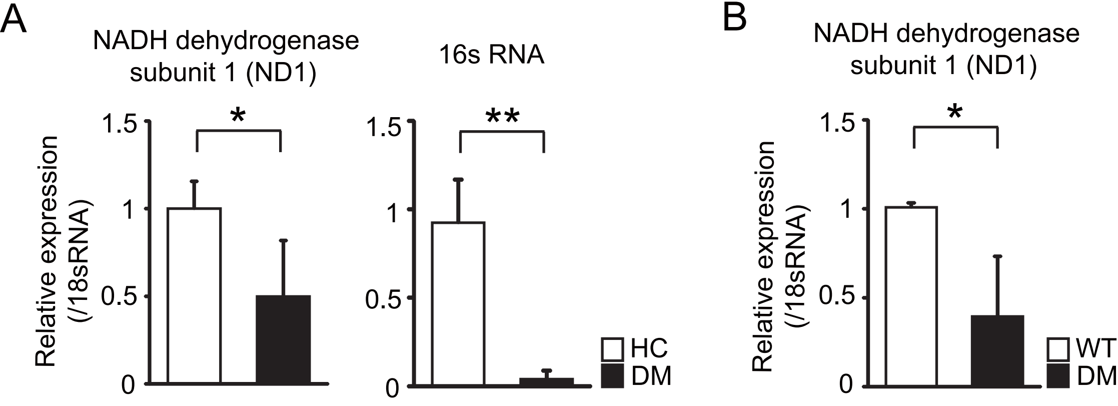

Appendix Figure S6

A

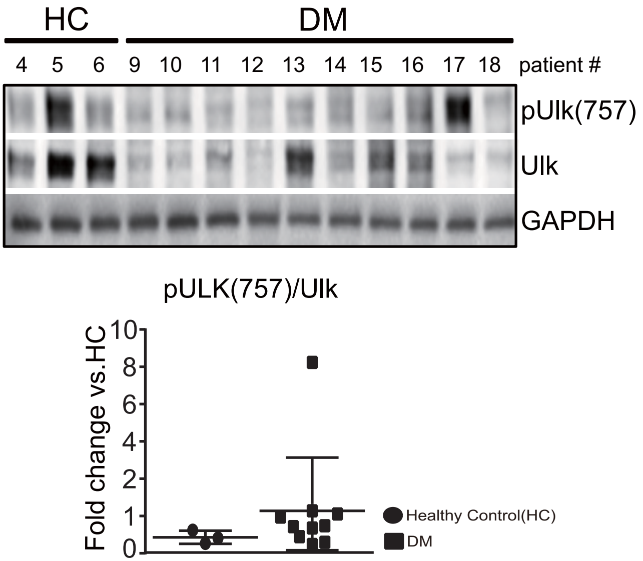

B

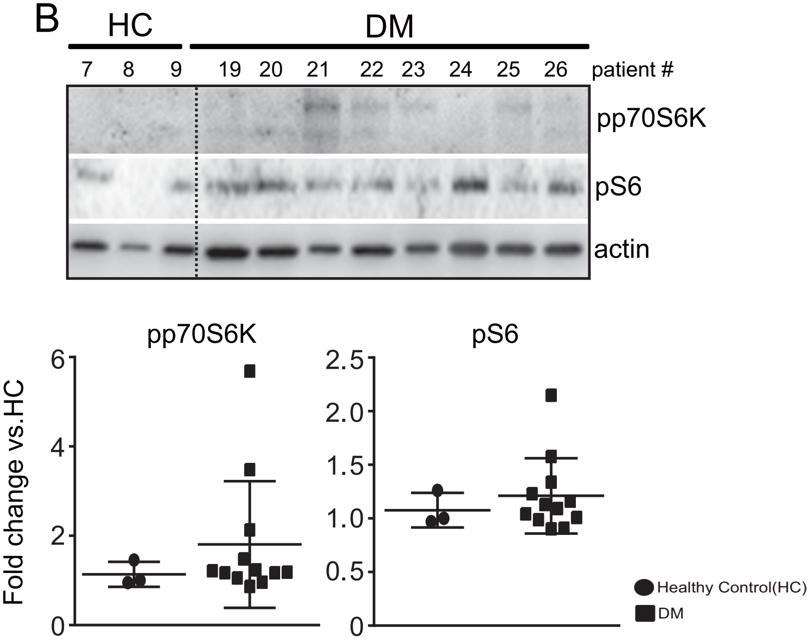

Appendix Figure S7

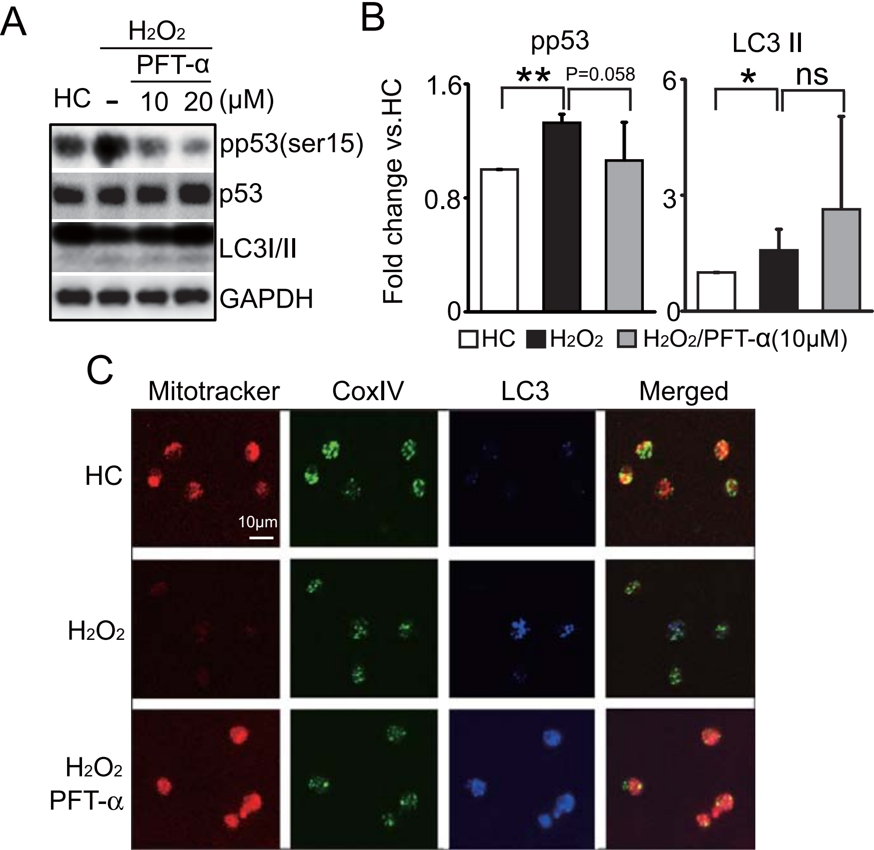

Appendix Figure S8

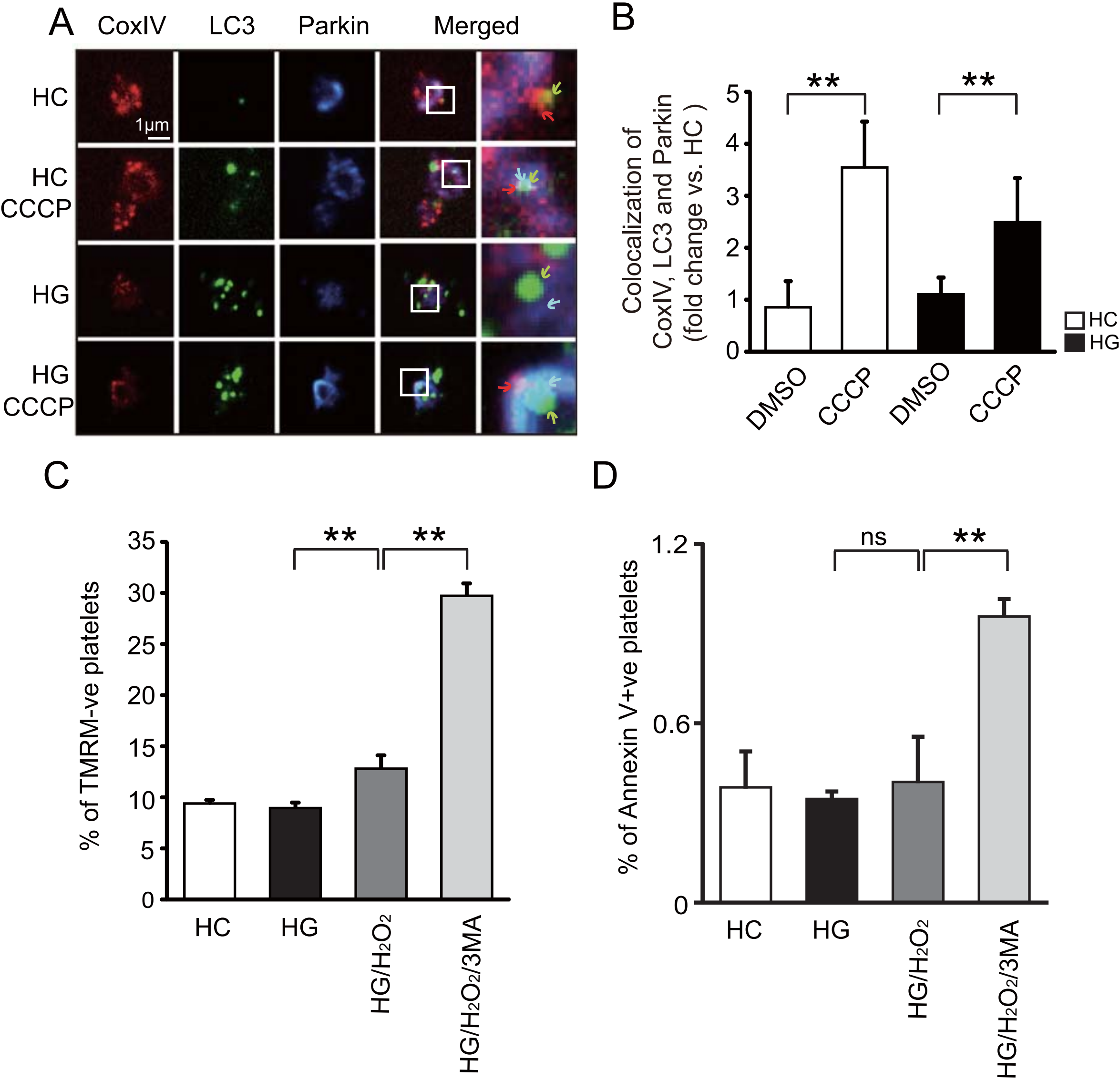

Appendix Figure S9

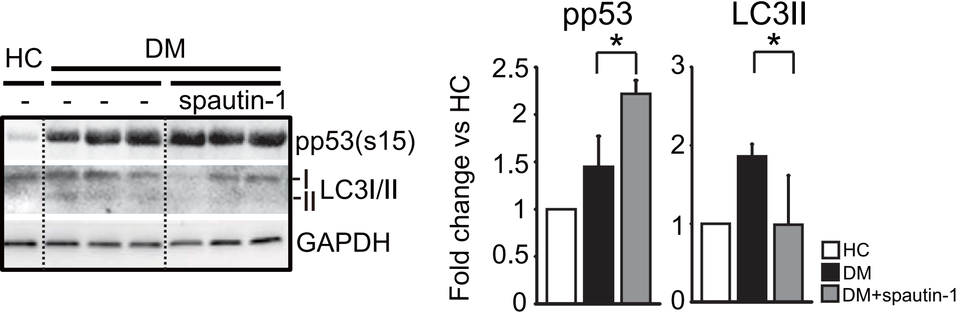

Appendix Figure S10

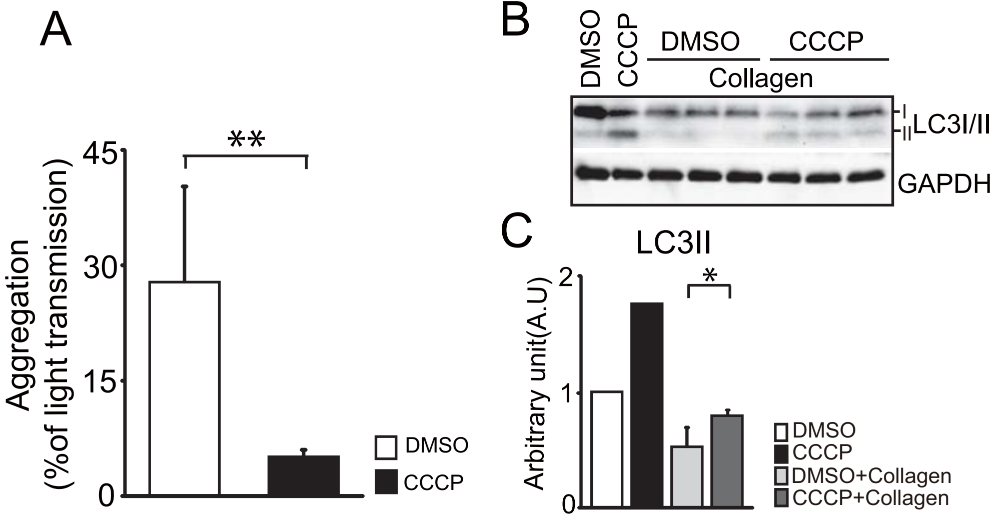

Appendix Figure S11

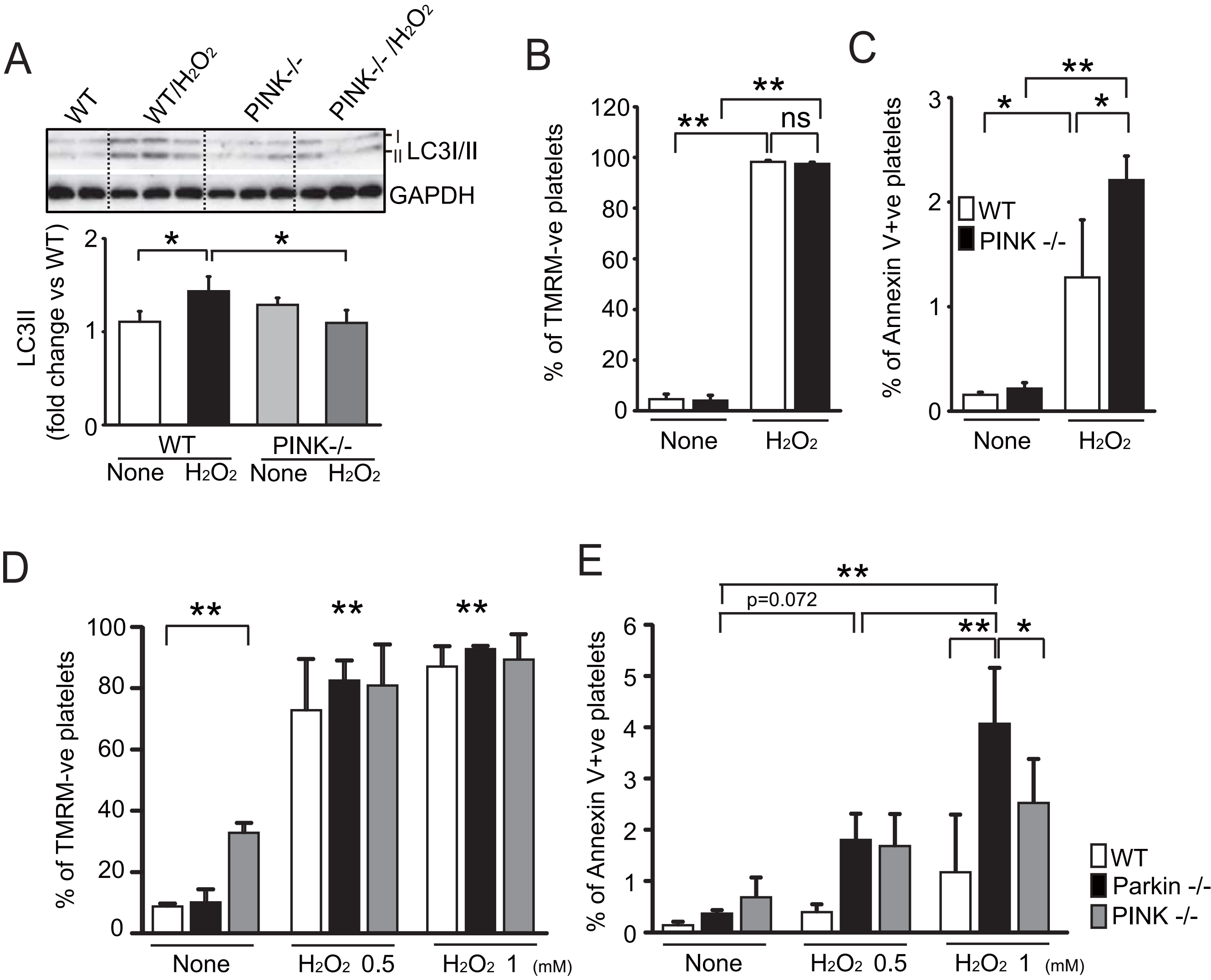

Appendix Figure S12

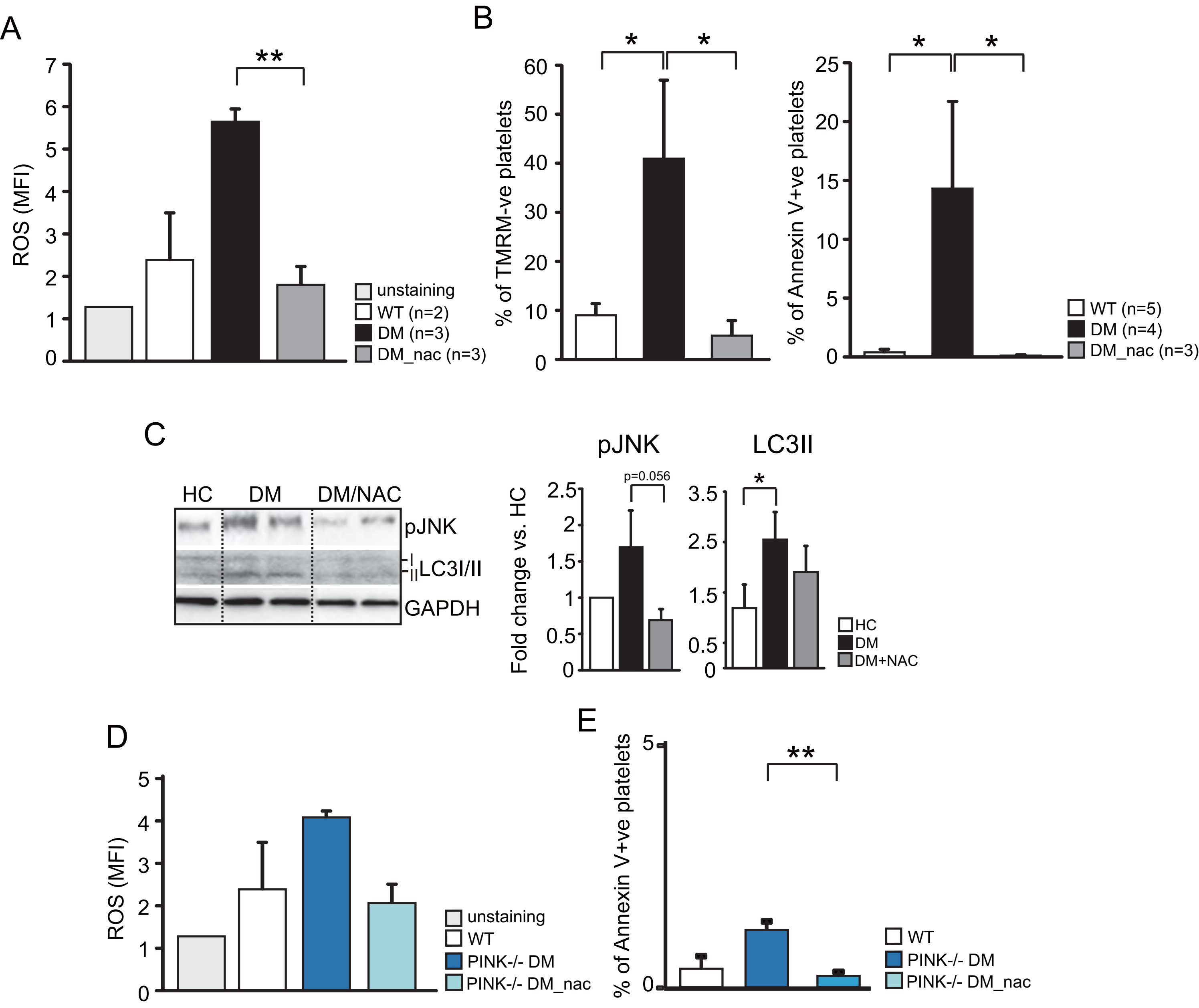

Appendix Figure S13

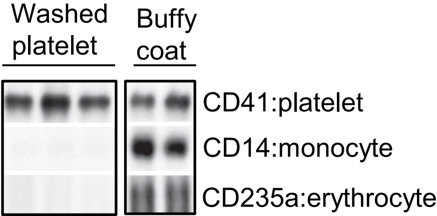

Supplement: Supplementary file 1 — Appendix [file EMMM-8-779-s001.pdf]
