# Supplementary figures and images for: Inducing mitophagy in diabetic platelets protects against severe oxidative stress
Source: EMBO Mol Med. 2016 May 24;8(7):779–95. doi: 10.15252/emmm.201506046 (PMC4931291; doi:10.15252/emmm.201506046)

Figure 1\_Source File

A

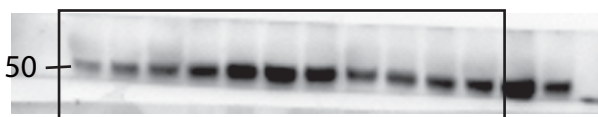

pp53 in HC and DM

C

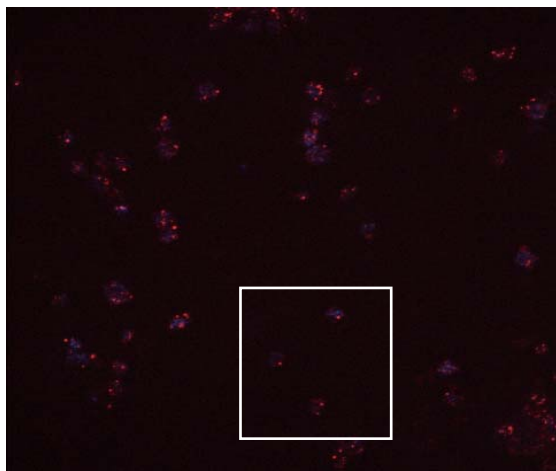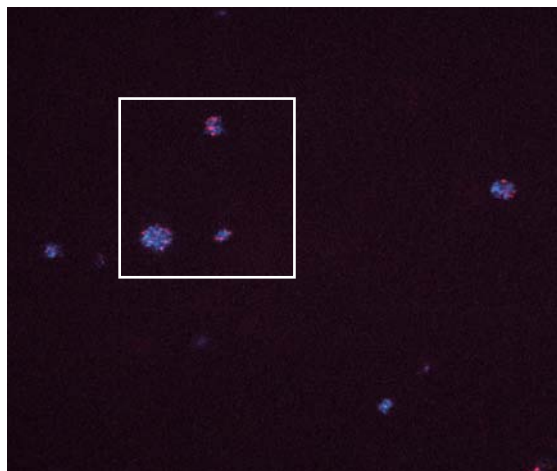

D

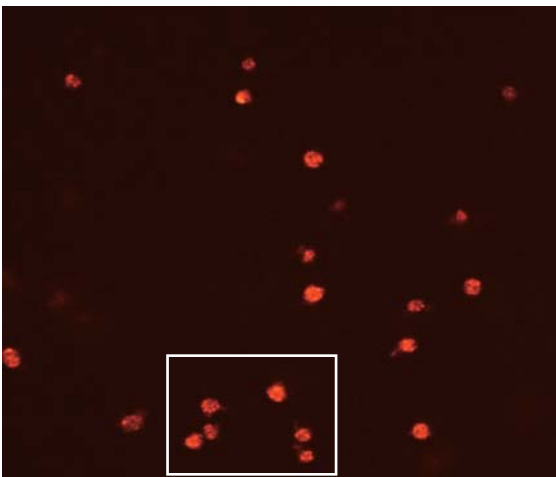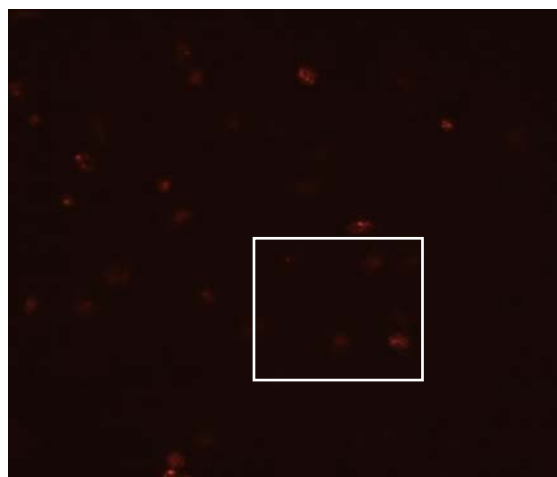

Supplement: Supplementary file 3 — Source Data for Figure 1 [file EMMM-8-779-s002.pdf]

Figure 2\_source file

A

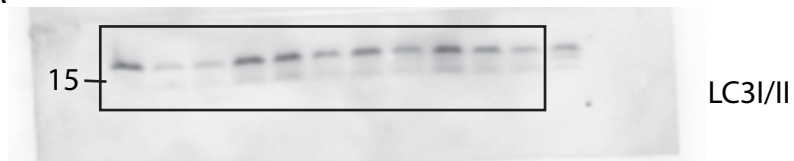

E, F, G

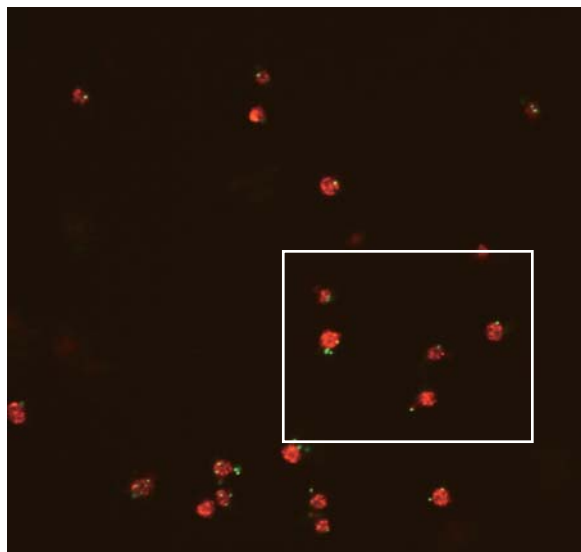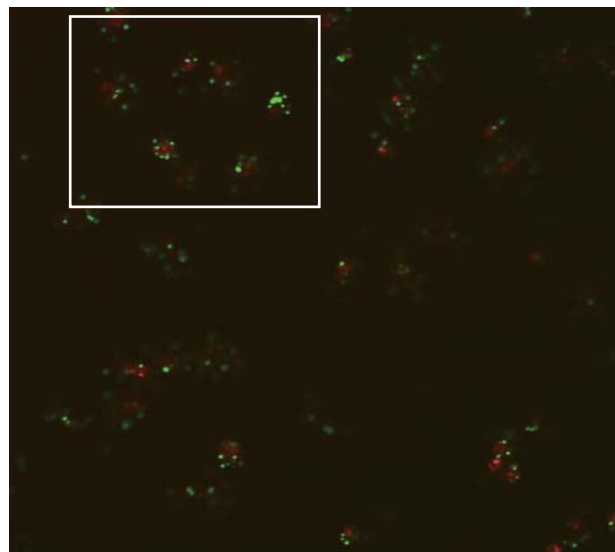

Supplement: Supplementary file 4 — Source Data for Figure 2 [file EMMM-8-779-s003.pdf]

A

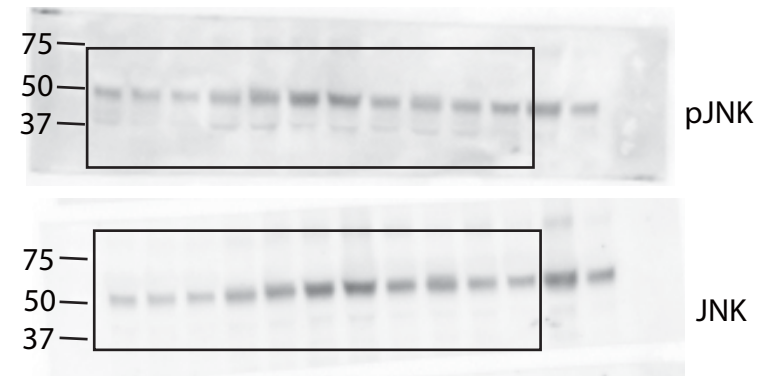

E

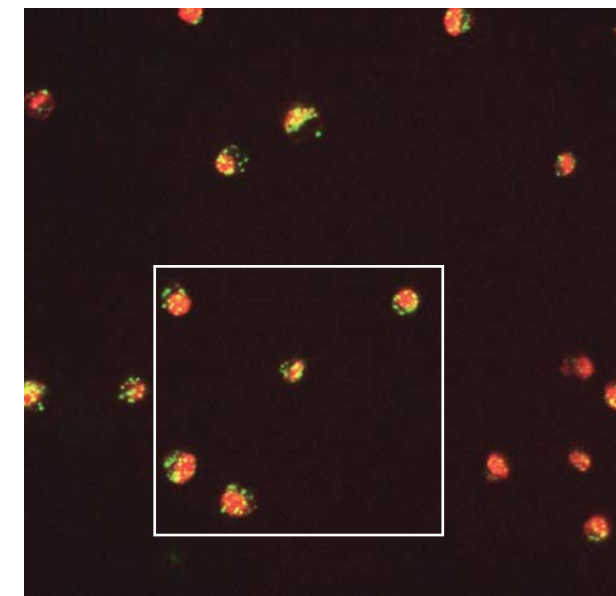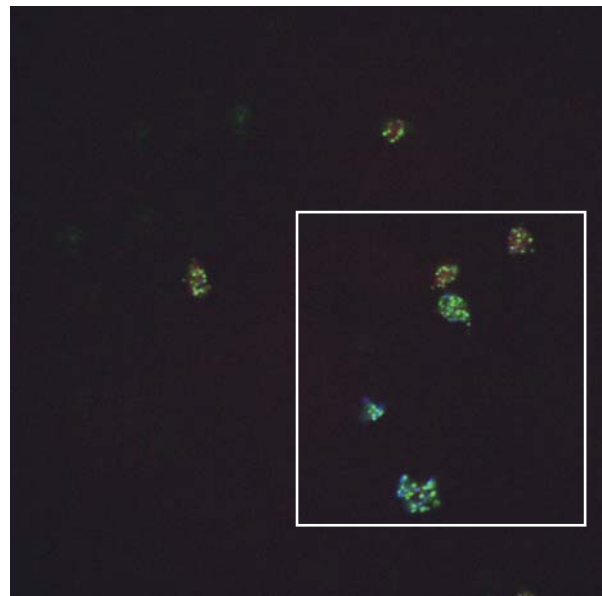

Supplement: Supplementary file 5 — Source Data for Figure 3 [file EMMM-8-779-s004.pdf]

F

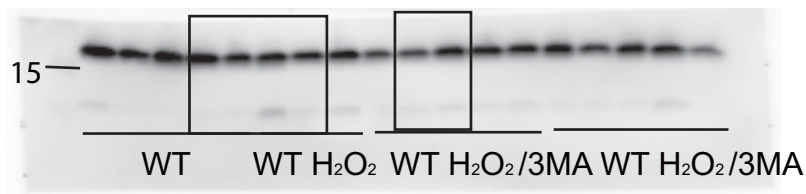

J

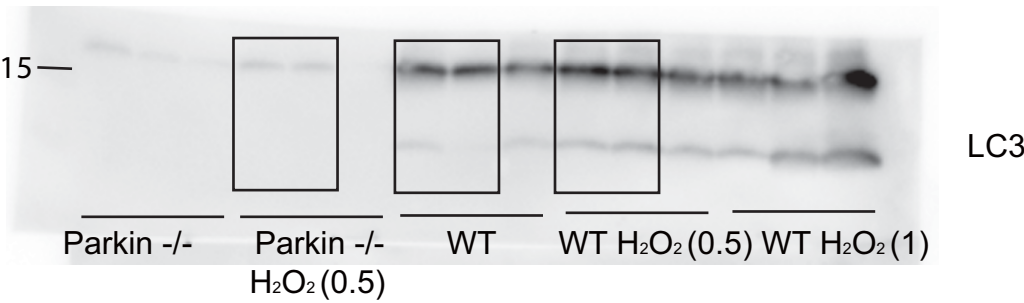

Supplement: Supplementary file 6 — Source Data for Figure 5 [file EMMM-8-779-s005.pdf]
